# Observation of Static Pictures of Dynamic Actions Enhances the Activity of Movement-Related Brain Areas

**DOI:** 10.1371/journal.pone.0005389

**Published:** 2009-05-06

**Authors:** Alice Mado Proverbio, Federica Riva, Alberto Zani

**Affiliations:** 1 Department of Psychology, University of Milano-Bicocca, Milan, Italy; 2 Institute of Bioimaging and Molecular Physiology, National Research Council (CNR), Milano-Segrate, Italy; James Cook University, Australia

## Abstract

**Background:**

Physiological studies of perfectly still observers have shown interesting correlations between increasing effortfulness of observed actions and increases in heart and respiration rates. Not much is known about the cortical response induced by observing effortful actions. The aim of this study was to investigate the time course and neural correlates of perception of implied motion, by presenting 260 pictures of human actions differing in degrees of dynamism and muscular exertion. ERPs were recorded from 128 sites in young male and female adults engaged in a secondary perceptual task.

**Principal Findings:**

Our results indicate that even when the stimulus shows no explicit motion, observation of static photographs of human actions with implied motion produces a clear increase in cortical activation, manifest in a long-lasting positivity (LP) between 350–600 ms that is much greater to dynamic than less dynamic actions, especially in men. A swLORETA linear inverse solution computed on the dynamic-minus-static difference wave in the time window 380–430 ms showed that a series of regions was activated, including the right V5/MT, left EBA, left STS (BA38), left premotor (BA6) and motor (BA4) areas, cingulate and IF cortex.

**Conclusions and Significance:**

Overall, the data suggest that corresponding mirror neurons respond more strongly to implied dynamic than to less dynamic actions. The sex difference might be partially cultural and reflect a preference of young adult males for highly dynamic actions depicting intense muscular activity, or a sporty context.

## Introduction

It is known that when actions by other individuals are observed, the somatosensory, motor and premotor cortices of the viewer resonate by activating a neural population mirroring the perceived actions [1]–[3]. In particular, metabolic activity is enhanced in response to human motion, reflecting somatotopic organization, in the premotor and parietal areas [4], the superior temporal cortex [5], the extra-striate (BA19/37), inferior parietal and cingulate cortex [6]. Recently, Gazzola and Keysers [7] provided indisputable evidence that observation of action activates not only the ventral premotor (BA6/44) and inferior parietal cortices, where mirror neurons have been found in monkeys, but also the dorsal premotor, supplementary motor, middle cingulate, somatosensory superior parietal, middle temporal cortices and the cerebellum. Many theories have suggested a possible role for mirror neurons in understanding the meaning and intentions of observed actions, learning by imitation [8], feeling empathy [9], formation of a ‘theory of mind’, and even the development of language [10]. Notwithstanding they have been widely documented in macaque monkey, mirror neurons research in humans is more problematic since assessing neural selectivity using non-invasive techniques is rather difficult [11]. The protocols used usually involve passive osservation vs. rest or execution vs. observation of movements, which sometimes is problematic since there are many other neurons (in addition to mirror neurons) in diverse cortical areas that increase their responses in a non selective manner during these tasks. The development of new experimental protocols will help increase our understanding of the role of the mirror system in the human mind.

In an electrophysiological study [12], EEGs were recorded while the participants observed human or non-biological movements. The results showed significantly higher activation in the primary motor and premotor cortex and supplementary motor area as well as the posterior parietal cortices during observation of biological movements, consistent with the mirror properties of cortical motor neurons.

Other studies have demonstrated that motor and premotor mirror neurons fire in response not only to viewing actual dynamic human actions [4] but also to dynamic information from static pictures of individuals captured in the midst of motion, i.e. so-called implicit motion [13].

So far, investigations have involved comparisons of the perception of dynamic actions vs. static bodies, or biological vs. non-biological movements. In the light of the available literature we wished to investigate the neural processing of implicit human motion further by recording brain potentials evoked by observing more or less effortful actions (e.g., jumping vs. washing hands), which are correlated with different degrees of muscular tension in the agents.

In this regard, a physiological study of perfectly still observers [14] has shown an interesting correlation between greater effortfulness of observed actions (which included weight-lifting, running, walking at increasing weight or speed) and increased respiration rate. In another study [15], mental simulation of the motor action of running on a treadmill at increasing speed provoked an increase in heart rate as well as changes in respiratory parameters. These changes were proportional to the degree of simulated effort. This result was replicated in a further experiment [16] that involved pedalling against a load at an increasing rate: the heart and respiratory rates increased with the pedalling rate. This facilitation may represent the neural basis for important functions such as imitation or learning by observation.

Overall, these data suggest that the perceived effortfulness of visually presented actions affects the autonomic response by increasing the heart and respiratory rates as a function of the perceived muscular effort. While little is known about the concomitant response of the central nervous system and related brain structures, it may be relevant that mental simulation of actions in perfectly still persons has been reported to activate central motor structures, including the lateral cerebellum, basal ganglia, premotor cortex and posterior parietal cortex [17]–[19].

**Figure 1:**
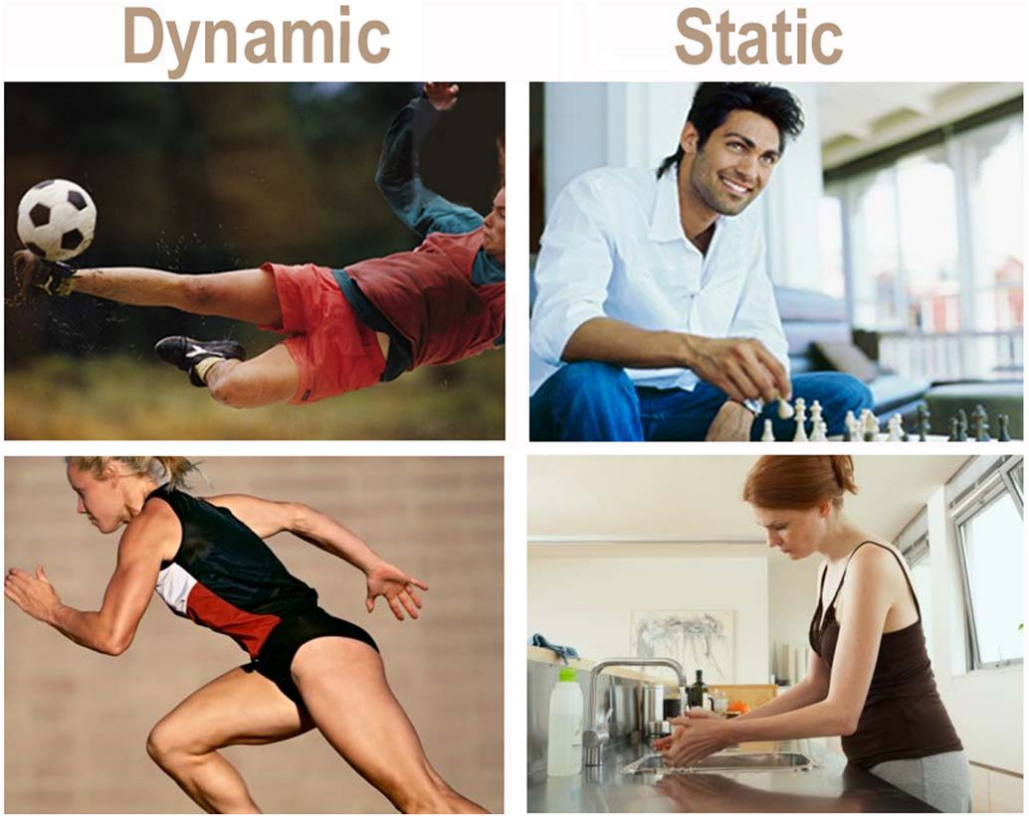
Some example of stimuli belonging to the two classes of actions balanced for body part depicted and sex of agent.

The overall aim of this study was to investigate the time course and neural correlates of implicit motion perception, by presenting human actions differing in degree of dynamism and muscular exertion. We hypothesized that the contrast between static and dynamic actions and their neural processing might shed some light on the neural processing of implicit motion perception and action representation. For this purpose, 260 static pictures of women and men engaged in simple dynamic and almost static actions (see some examples in Fig. 1) were presented to right-handed university students, who did not practise sporting disciplines at competitive levels, while they were engaged in a secondary perceptual task.

## Results

**Figure 2:**
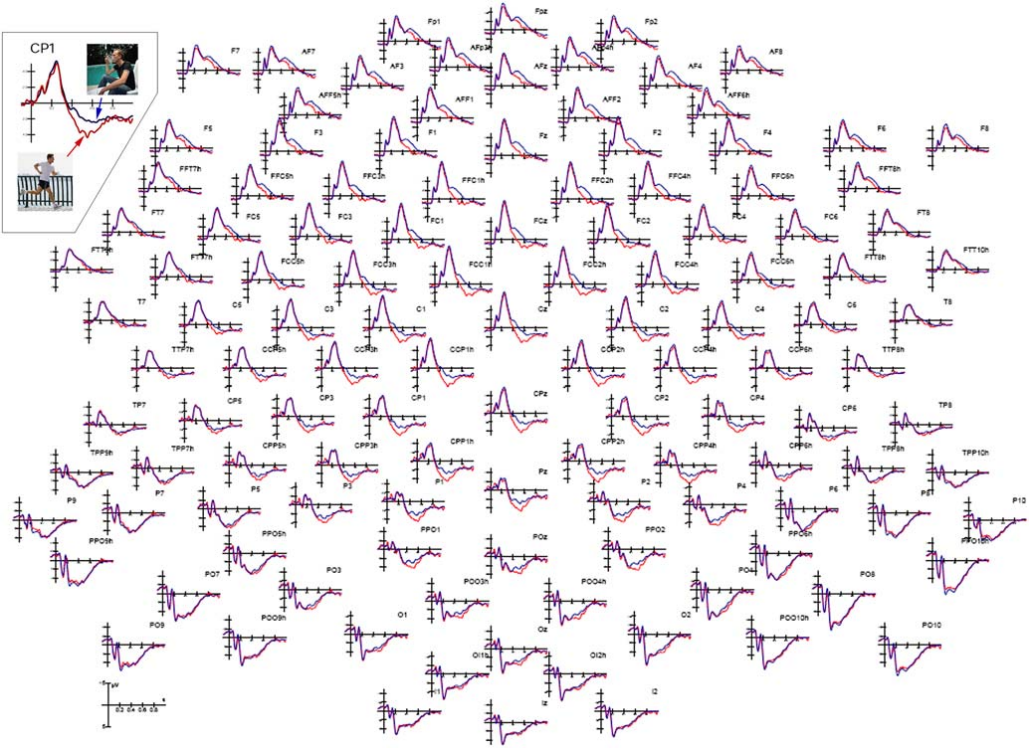
Grand-average ERP waveforms (N = 23) recorded over all scalp sites as a function of action type.

Fig. 2 shows the grand average ERP waveforms recorded at anterior and posterior scalp sites as a function of the motor content of the pictures.

**Figure 3:**
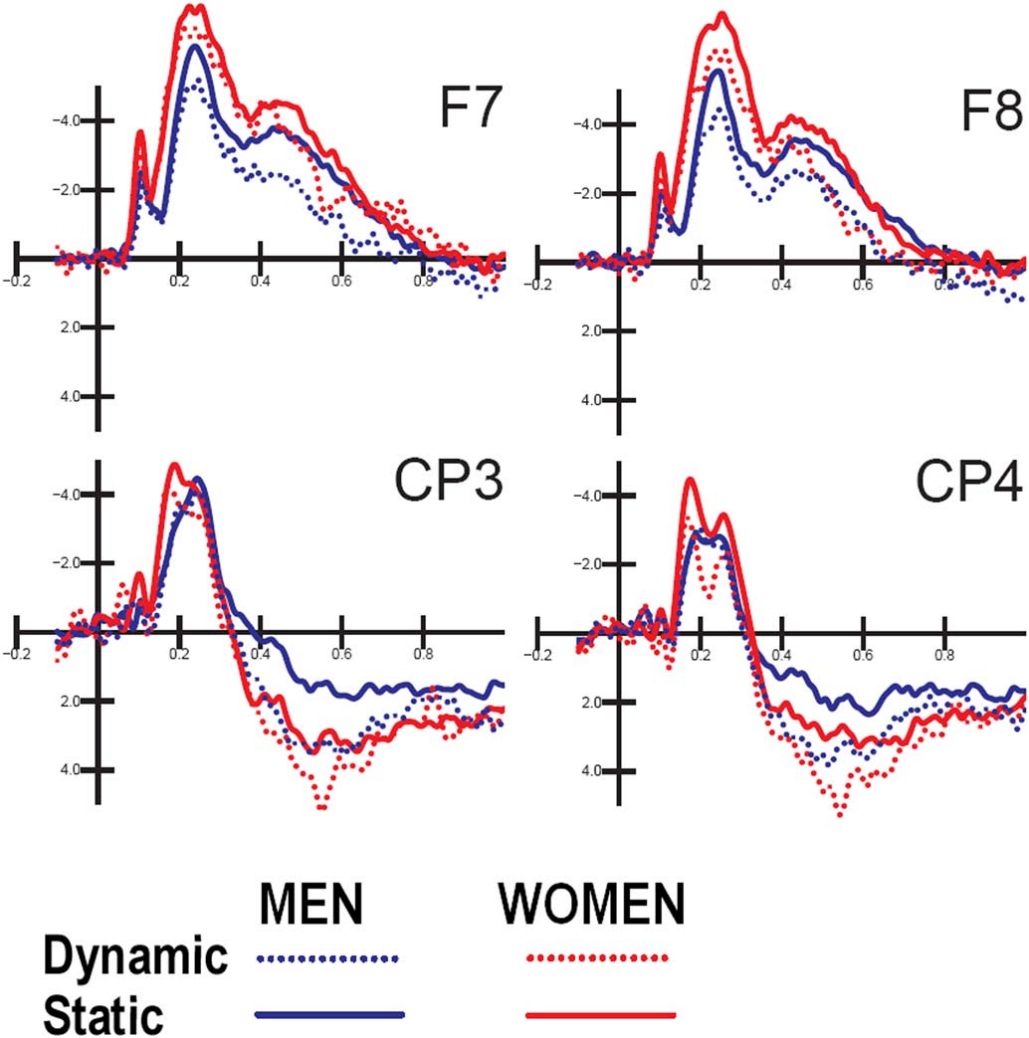
ERP waveforms recorded over left and right inferior/frontal and centro-parietal sites as a function of viewer's sex and action type. Motor content evidently has a greater effect in the male brain, which appeared more responsive to the representation of vibrant and intense muscular activity.

A long-lasting centro/parietal deflection or late positivity (LP) is observable, which is much larger to dynamic than static actions (implied motion). The effect of the action's content was also very conspicuous at frontal sites, where the LP was smaller. ANOVA showed that the motor content factor was significant (F1,21 = 29.6; p<0.000025), with a larger LP to dynamic (−0.003 µV, SD = 0.94) than static (−0.93 µV, SD = 0.92) actions. The LP was larger at centro/parietal (2.09 µV) than inferior frontal (−3.02 µV) sites, but the motor content effect was similar across sites, as indicated by the lack of interaction of electrode×motor content. Interestingly, the action's content was more significant in men (eff: 0.17; not eff.: −1.12 µV) than women (Eff.: −0.18; not eff: −0.73 µV), as shown by the interaction of the latter factor×sex, and by relative post-hoc comparisons. This effect is displayed in waveforms in Fig. 3.

**Figure 4:**
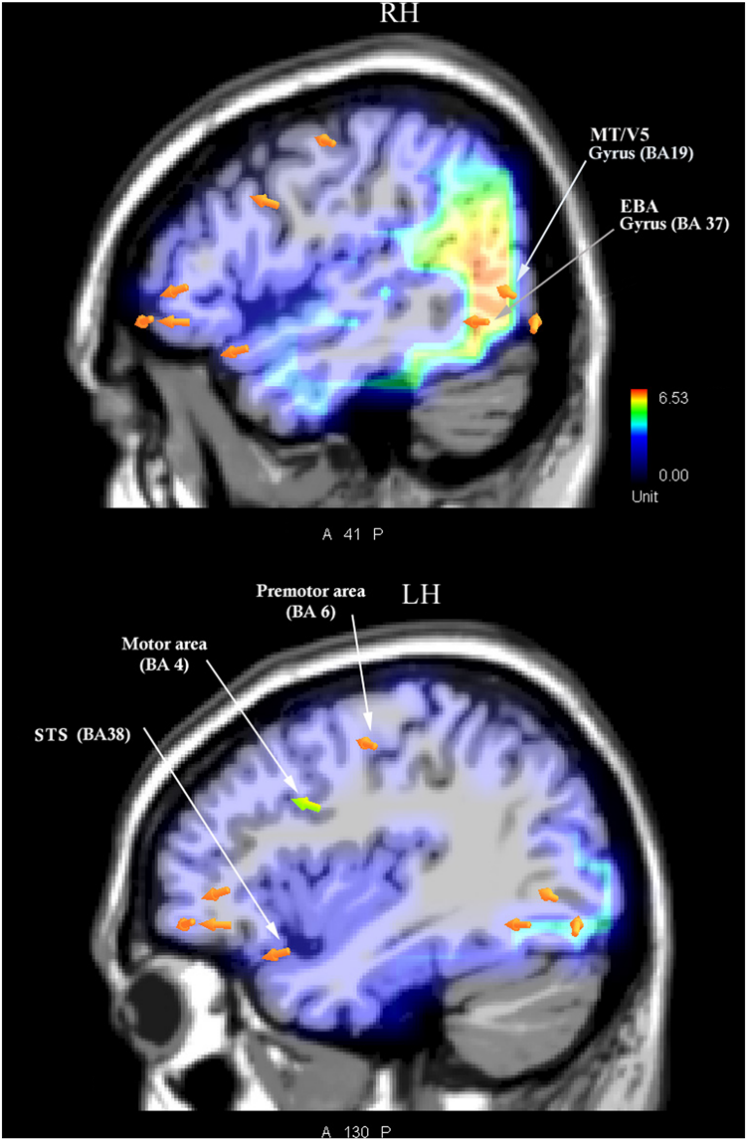
LORETA inverse solution displaying the neural generators of the LP effect related to action dynamism. LORETA was computed on the difference wave obtained by subtracting ERPs to static actions from ERP to dynamic actions in the time window 380–430 ms, corresponding to the ascending phase of the LP.

**Figure 5:**
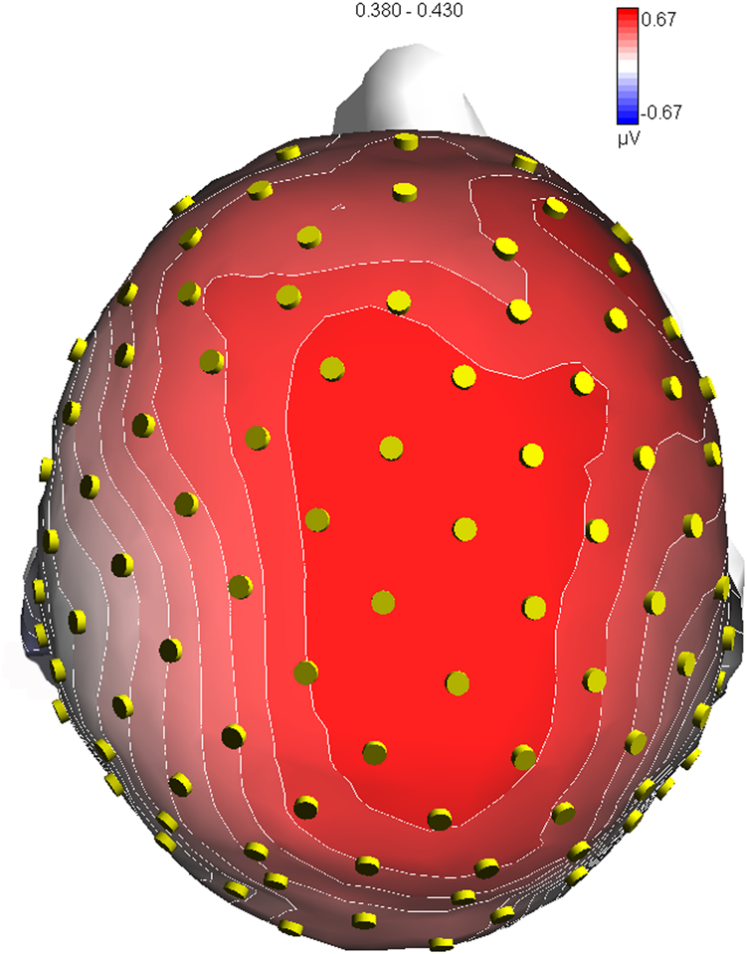
Top view of isocontour topographic voltage map relative to the difference wave dynamic – static in the 380–430 ms time window.

In order to locate the possible neural source of the motor content and effortfulness effect for implied motion perception, two separate swLORETA source reconstructions were performed on the difference waves obtained by subtracting the ERPs to static from those elicited by dynamic pictures in two adjacent time windows, 380–430 and 430–480 ms. The resulting neural activity, visible in Fig. 4, might reflect the activation of neural circuits subserving both implicit motion perception and action representation. Fig. 5 shows the scalp voltage topography of the LP difference on which the source analysis was based. The neural generators relevant to this contrast (listed in Table 1) in the first window are the left and right MT/V5 areas (BA19), the left inferior temporal gyrus (IT, BA37), the left premotor (BA6) and motor (BA4) areas, the left superior temporal gyrus (STG, BA38), the right anterior cingulate, and the left and right middle frontal gyrus (BA47/10). The second time window showed persistence of the greater activation of the left IT gyrus and right STG (BA38) for processing dynamic vs. static actions, and also of the right cingulate, left superior and right inferior frontal gyri.

**Table 1:** Tailarach coordinates corresponding to the intracranial generators explaining the difference voltages related to effortful/dynamic minus less effortful actions in the 380–430 and 430–480 ms time windows, according to swLORETA (ASA) [31]; grid spacing = 5 mm, estimated SNR = 3.

380–430 ms							
Magnit	T-x [mm]	T-y [mm]	T-z [mm]	H	Lobe	Area	BA
5.19	−48.5	−76.2	−11.7	LH	Temp	Fusiform gyrus	19
6.53	50.8	−67.1	−3.5	RH	Temp	Inferior Temporal gyrus	19
5.05	−58.5	−55.9	−10.2	LH	Temp	Inferior Temporal gyrus	37
2.54	−28.5	−14.4	45.5	LH	Front	Precentral gyrus	6
2.18	−38.5	2.4	29.4	LH	Front	Precentral gyrus	4
2.84	−48.5	17.2	−11.9	LH	Temp	Superior Temporal gyrus	38
2.42	11.3	35.3	5.3	RH	Limbic	Anterior Cingulate	24
2.80	−48.5	36.3	−3	LH	Front	Middle Frontal gyrus	47
3.38	40.9	46.3	−2.3	RH	Front	Middle Frontal gyrus	10
**430–480 ms**							
7.67	50.8	−57.9	5.6	RH	Temp	Middle Temporal gyrus	21
7.61	−58.5	−55.9	−10.2	LH	Temp	Inferior Temporal gyrus	37
5.84	11.3	−40.6	34	RH	Limbic	Cingulate gyrus	31
3.80	−38.5	46.3	−2.3	LH	Front	Inferior Frontal gyrus	10
3.65	21.2	56.3	−1.6	RH	Front	Superior Frontal gyrus	10
2.61	50.8	17.2	−11.9	RH	Temp	Superior Temporal gyrus	37

## Discussion

Our results indicate that even in the absence of explicit stimulus motion, observation of static photographs of human actions with implied motion produces a clearly greater cortical activation than observation of static images of less dynamic actions. ERP analysis showed that the motor content of the photographs exerted a strong effect, with a much larger positivity at all scalp sites (ranging from 350 to 600 ms in latency) in response to dynamic/effortful than to less effortful (static) actions. Since the pictures were balanced for other types of perceptual parameters except the degree of implicit biological motion represented, and the observers were engaged in a secondary target detection task, the data suggest a strong effect of implied motion on the amplitude of the ERPs.

The swLORETA linear inverse solution computed on the dynamic-minus-static difference wave in the time window corresponding to the ascending phase of late positivity (380–430 ms) showed activation of a series of regions belonging to the action and motion representation systems, namely: V5/MT, EBA, STS, premotor and motor areas, and cingulate IF cortex. In the next temporal window (430–480) V5/MT, motor and premotor areas were no longer activated (more to dynamic than static pictures), while cingulate activation was increased along with the inferior frontal and orbitofrontal cortices, possibly suggesting a switch from a sensory-motor code for action representation to a more abstract cognitive/affective code for representing visual information.

The activation of motor areas when viewing implicit biological motion is fully consistent with previous literature [13] on implied body actions, providing evidence of the brain's ability to extract motion information from static images. In particular, Urgesi and coworkers, using single-pulse transcranial magnetic stimulation, found that the mere observation of static snapshots of hands suggesting a pincer grip action induced greater corticospinal excitability than observation of resting, relaxed hands, or hands suggesting a completed action.

In our study, the greater activation of BA19 for dynamic than for static actions indicates involvement of area V5/MT, which has been shown to respond not only to real motion [20] but also to implicit [21], [22] and illusory [23], [24] motion. Similarly, the greater activation of the STG, cingulate and extrastriate body area (EBA) regions for processing more dynamic actions suggests that they have a role in biological motion and action representation, as indicated by many studies on the mirror neuron system (MNS) in humans [25]–[28].

The EBA, located in the lateral occipitotemporal cortex (BA37), was first reported to respond selectively to visual images of human bodies or body parts [29]. Later it was shown that the EBA responds not only during the perception of other people's body parts, but also during goal-directed movements of the observer's body parts [26]. fMRI studies using point-light animations of biological motion showed that viewing biological motion selectively activates the posterior superior temporal sulcus (STSp) [27], [28]. The role of the EBA in the perception of body motion is not really clear, since for example in one of these studies [28] it was found that BOLD responses to whole bodies in motion and to images of stationary, headless bodies were equivalent in the EBA but not STS regions, which were selectively driven by the dynamics of the human form.

In our study, the finding of brain regions devoted to motion and action processing supports the hypothesis that implied motion was the crucial factor in determining a difference in brain activation between the two conditions. However, it cannot be excluded that other possible confounding factors such as attraction, emotional valence, preference and interest might have contributed to determine a greater amplitude of LP to dynamic vs. static pictures, or to induce specific sex differences. At this regard, two additional LORETAs performed separately in women and men relative Tailarach coordinates of significant activations are reported as Supplementary material in Fig. S1 and Table S1. Overall, the results suggest that men exert a higher degree of neuronal mirror activity to movement related pictures (either for evolutionary and/or cultural reasons), whereas women showed a strong visual interest for all human figures [30].

Overall, the present electrophysiological and swLORETA source reconstruction findings provide evidence of greater activation of the EBA, V5/MT, STG (BA38), motor (BA4) and premotor (BA 6) areas in response to effortful (dynamic) than static actions, thus suggesting a stronger response of the corresponding mirror neurons to implicit motion [13]. This pattern of results may provide the cortical counterpart to the autonomic response described in previous physiological literature [14]–[16], [19], suggesting an interesting correlation in perfectly still observers between greater effortfulness of observed actions and increased respiration and heart rates.

The sex difference in cortical sensitivity to dynamic actions, consisting in a finer discriminative response of the LP in males than females, might possibly be ascribed to a difference of cultural origin (but probably not entirely). In any case, it might reflect a men preference for highly dynamic actions depicting intense muscular activity, or a sporty context.

## Materials and Methods

### Participants

Twenty-three healthy right-handed Italian University students (12 males and 11 females) were recruited for this experiment. Their ages ranged from 20 to 35 years (mean = 24.79; DS = 3.15). All had normal or corrected-to-normal vision and reported no history of neurological illness or drug abuse. None of the participants practised a sporting discipline at competitive level (except for one girl who had practised agonistic swimming at an earlier age). Many of the subjects (with no notable sex difference) practised sporting activities (volleyball, soccer, swimming, dancing) once or twice a week to keep fit. Experiments were conducted in accordance with the Declaration of Helsinki and with the understanding and the written consent of each participant.

### Stimuli

Two hundred and sixty ecological colour pictures representing persons differing in number, age and gender, engaged in relatively static (not effortful) or dynamic (effortful) actions (see some examples in Fig. 1), were presented in the central visual field of a PC screen, randomly mixed with 44 neutral scenarios lacking visible people.

The pictures were balanced across classes for gender (males, females), age (children, adults), number of persons (one, more than one), body parts depicted (full-length, half-length, close-up), picture size (11°27′53″ in length and 8°35′55″ in height) and average luminance (143.66 Footlambert). Stimuli were presented randomly mixed for 1500 ms with an ISI of 1800–1900 ms on a grey background.

Effortful actions included pictures of persons engaged in rather dynamic actions such as running, jumping, exercising, shovelling snow, pulling, pushing or carrying something heavy. Less effortful actions portrayed persons engaged in relatively less muscularly fatiguing activities such as reading, painting, having a bath, eating, doing manual work while seated, speaking on the phone, etc.

Pictures were selected according to the criterion that they belonged to the typical human repertoire, and categorized by three independent judges on the basis of the dynamicity of implied motion as: 1) Static if the body was basically still except of some not effortful arm movement, and 2) Dynamic if the body (including legs) was in motion, and the action was effortful showing muscular tension. Slides characterized by an intermediate level of action dynamicity were discarded.

### Procedure

The participants, seated comfortably in a dimly lit, electrically and acoustically shielded room, faced a window behind which a high resolution VGA computer screen was positioned 80 cm from their eyes. A small bright dot (1 mm in size) located at the centre of the screen served as a fixation point to minimize eye movements. The subjects were instructed to fixate the centre of the screen and to avoid any eye or body movements during the recording session. The task consisted in signalling the rare presentation of a natural landscape without visible humans (44 in all) by pressing a button as accurately and rapidly as possible with the index finger of the left or right hand. The two hands were used alternately during the recording session, and the hand and sequence order were counterbalanced across subjects.

### EEG recording and analysis

The EEG was continuously recorded from 128 scalp sites at a sampling rate of 512 Hz. Horizontal and vertical eye movements were also recorded. Linked ears served as the reference lead. The EEG and electro-oculogram (EOG) were amplified with a half-amplitude band pass of 0.016–100 Hz. Electrode impedance was kept below 5 kΩ. The artefact rejection criterion was peak-to-peak amplitude exceeding 50 µV, and the rejection rate was ∼5%. ERPs were averaged off-line from −100 ms before to 1000 ms after stimulus onset.

The mean amplitude of late positivity was measured at centroparietal sites (CP3 e CP4) and frontal sites (F7, F8) in the time window 380–480 ms.

ERP data were subjected to multifactorial repeated-measures ANOVA with one between-groups and three within-group factors of variability. These latter factors were the motor content of the action (dynamic, static), electrode (frontal, centro/parietal) and hemisphere (left, right). Multiple comparisons of means were performed by post-hoc Tukey tests. The between-groups factor was the sex of the participants (men, women).

## Supporting Information

Table S1 (0.05 MB DOC)

Figure S1 Sex differences: LORETA inverse solution displaying the neural generators of the LP effect related to action dynamism. LORETA was computed on the difference wave obtained by subtracting ERPs to static actions from ERP to dynamic actions in the time window 380–430 ms, separately for women (left) and men (right).
(3.53 MB TIF)
