# Supplementary material for: Observation of Static Pictures of Dynamic Actions Enhances the Activity of Movement-Related Brain Areas
Source: PLoS One. 2009 May 6;4(5):e5389. doi: 10.1371/journal.pone.0005389 (PMC2671843; doi:10.1371/journal.pone.0005389)
Supplement: Table S1 — (0.05 MB DOC) [file pone.0005389.s001.doc]

**Table S1**

## Tailarach coordinates corresponding to the intracranial generators explaining the difference voltages related to dynamic minus static actions in the 380-430 ms time windows, separately for men and women (swLORETA (ASA) [25]; grid spacing = 5 mm, estimated SNR = 3).

The results support the hypothesis of a male greater activation in response to dynamic implied motion. Electromagnetic signals were stronger in cortical regions belonging to the human mirror system in men: namely the superior parietal cortex (BA7), the STS and the anterior prefrontal cortex (BA10). In the female brain the different voltage was much smaller and localized more posteriourly along the ventral stream in regions possibly corresponding to EBA, MT/V5, left IT gyrus and prefrontal cortex (BA11).

**MEN**

| **Magnit** | **T-x [mm]** | **T-y [mm]** | **T-z [mm]** | **H** | **Lobe** | **Area** | **BA** |
| --- | --- | --- | --- | --- | --- | --- | --- |
| 11 .19 | 11 .3 | -51 .7 | 42 | R | P | Precuneus | 7 |
| 11 .10 | 31 | -51 .7 | 42 | R | P | Superior Parietal Lobule | 7 |
| 6 .87 | -28 .5 | 56 .3 | -1 .6 | L | F | Superior Frontal Gyrus | 10 |
| 6 .11 | 1 .5 | 57 .3 | -9 | R | F | Medial Frontal gyrus | 10 |
| 4 .75 | -38 .5 | -28 .5 | 17 .1 | L | T | Superior Temporal Lobule | 41 |
| 11 .19 | 11 .3 | -51 .7 | 42 | R | P | Precuneus | 7 |
| 11 .10 | 31 | -51 .7 | 42 | R | P | Superior Parietal Lobule | 7 |
| 6 .87 | -28 .5 | 56 .3 | -1 .6 | L | F | Superior Frontal Gyrus | 10 |
| 6 .11 | 1 .5 | 57 .3 | -9 | R | F | Medial Frontal gyrus | 10 |

**WOMEN**

| 7 .62 | 40 .9 | -79 .2 | 12 .7 | R | O | Middle Occipital Gyrus | 19 |
| --- | --- | --- | --- | --- | --- | --- | --- |
| 4 .81 | -8 .5 | 0 .4 | 47 .2 | L | Limbic | Cingulate Gyrus | 24 |
| 4 .05 | -58 .5 | -55 | -17 .6 | L | T | Fusiform Gyrus | 37 |
| 4 .00 | -48 .5 | -33 .7 | -23 .6 | L | T | Fusiform Gyrus | 20 |
| 3 .51 | -38 .5 | -15 .3 | -29 .6 | L | T | Inferior Temporal gyrus | 20 |
| 3 .11 | 1 .5 | 38 .2 | -17 .9 | R | F | Medial Frontal Gyrus | 11 |
