# Supplementary figures and images for: Observation of Static Pictures of Dynamic Actions Enhances the Activity of Movement-Related Brain Areas
Source: PLoS One. 2009 May 6;4(5):e5389. doi: 10.1371/journal.pone.0005389 (PMC2671843; doi:10.1371/journal.pone.0005389)

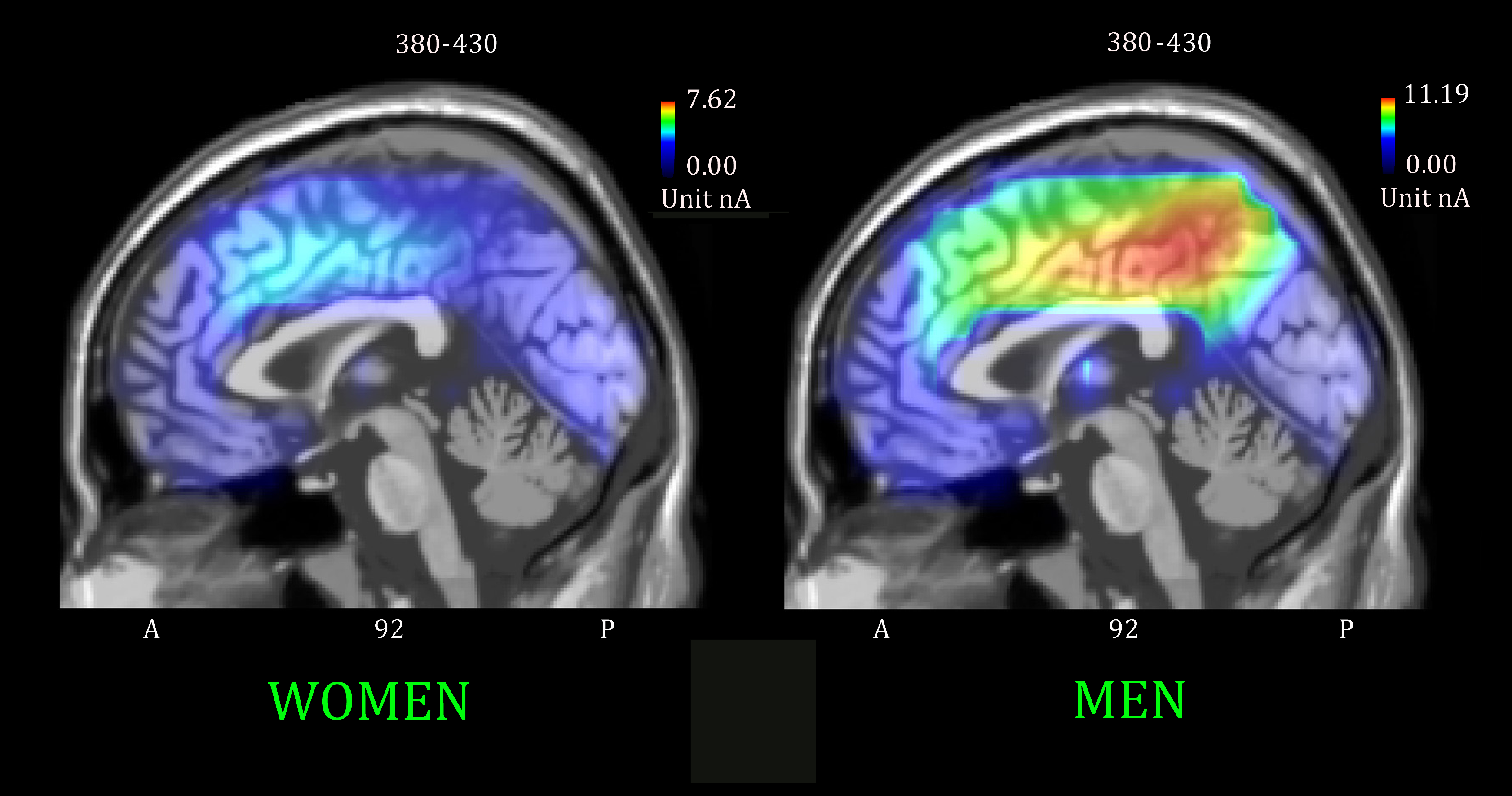

Supplement: Figure S1 — Sex differences: LORETA inverse solution displaying the neural generators of the LP effect related to action dynamism. LORETA was computed on the difference wave obtained by subtracting ERPs to static actions from ERP to dynamic actions in the time window 380–430 ms, separately for women (left) and men (right). (3.53 MB TIF) [file pone.0005389.s002.tif]
